# An efficient method for evaluating the lead equivalence of x‐ray radiation protective equipment using an analytical spectrum model

**DOI:** 10.1002/mp.70421

**Published:** 2026-04-03

**Authors:** Sewa Surdashi, Aseel Aziz, Shahla Mobini Kesheh, Jörgen Scherp Nilsson, Artur Omar

**Affiliations:** ^1^ Medical Radiation Physics and Nuclear Medicine Karolinska University Hospital Stockholm Sweden; ^2^ Department of Clinical Science, Intervention and Technology Karolinska Institutet Stockholm Sweden

**Keywords:** lead equivalence, radiation protection, radiation protective equipment

## Abstract

**Background:**

X‐ray radiation protective equipment is essential for ensuring the safety of medical staff. It is therefore important to verify its effectiveness, including confirming the specified lead equivalence (${\mathrm{Pb}}_{\mathrm{eq}}$), as it is a recognized standard protective value. Current methods require multiple comparative measurements with reference lead sheets, rendering the process laborious, susceptible to errors, and challenging to apply across a large medical facility with diverse protective equipment.

**Purpose:**

To introduce an efficient method for evaluating lead equivalence based on a computational model involving analytical spectrum modeling.

**Methods:**

The method consists of measuring the transmission of the protective equipment and then translating it into a lead‐equivalent thickness using a computational model. In this work, an example implementation is presented utilizing the SpekPy toolkit for spectrum modeling. To validate the method, it was used to estimate the thickness of high‐purity lead sheets with known thicknesses (0.1–1.0‐mm Pb). Furthermore, its application is demonstrated for two lead‐free aprons (0.25‐ and 0.35‐mm ${\mathrm{Pb}}_{\mathrm{eq}}$), a lead‐vinyl apron (0.5‐mm ${\mathrm{Pb}}_{\mathrm{eq}}$), a lead–acrylic and a lead–plywood mobile screen (0.5‐ and 1.0‐mm ${\mathrm{Pb}}_{\mathrm{eq}}$). Because the approach is based on measuring the transmission utilizing the primary x‐ray tube beam (rather than scatter from a phantom), Monte Carlo (MC) simulations were performed to identify x‐ray tube settings that reproduce clinically relevant scatter beams. Scatter spectra were simulated for different scatter angles (45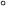, 90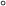, 135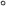), tube voltages (60–120 kV), and filtration (0.1–1 mm added copper). Analytical primary spectra were then matched to scatter spectra in terms of first and second tenth‐value layer (TVL) thicknesses in lead.

**Results:**

The method is accurate to within approximately 3% and is suitable for both narrow and broad beams. For broad beams, it is necessary to scale the measured transmission by the buildup factor for lead, as the analytical spectrum model does not account for scatter. This factor, which transfers broad‐beam air kerma to narrow‐beam air kerma, ranges from 1.0 to 1.5 for 50–120‐kV beams incident upon lead sheets with thicknesses of 0.1–1.0 mm. Without this factor, the lead equivalence can be underestimated by up to 28%. Using the method developed, it was found that the effectiveness of lead‐free aprons decreases by up to 20% for high‐kV and high‐filtration beams, while other equipment investigated agreed more closely with specifications. The MC simulations of scatter spectra indicated that scatter beams are generally softer than primary beams, with a reduction in TVL by up to 54% (average of 25%). The entire range of scatter‐mimicking primary beams can be realized with tube voltages 50–100 kV and less than 0.3 mm added copper filtration.

**Conclusions:**

The method developed can accurately convert measured transmission into lead equivalence using a computational model, which eliminates the need to handle physical lead sheets. The transmission can be measured using recommended scatter‐mimicking x‐ray tube beams, derived here for a broader range of scatter angles and clinical beams with higher filtration than has previously been considered.

## 1 INTRODUCTION

Diagnostic and interventional x‐ray imaging procedures pose radiation exposure risks that affect not only patients but also medical staff due to radiation scattered from patients. Recognizing this hazard, the International Commission on Radiological Protection has endorsed practices aimed at reducing exposure through the use of appropriate radiation protective equipment.^1^

Among the most widely adopted protective equipment are shielding garments, such as aprons, with a specified lead equivalence of 0.25, 0.35 or 0.5 mm. These garments are essential for mitigating radiation exposure faced by medical staff who work in close proximity to patients during x‐ray examinations, including image‐guided interventions that may expose staff to radiation doses exceeding occupational dose limits.^2^ In addition to protective garments, table‐mounted shields, as well as mobile and ceiling‐suspended lead–acrylic screens with a specified lead equivalence between 0.5 and 1 mm, are common in interventional and hybrid operating rooms.^3^, ^4^ Mobile lead–acrylic screens are also employed in intensive care units where portable x‐ray equipment is in use. The adoption of such screens has grown in recent years, partly influenced by research indicating a heightened risk of radiation‐induced eye lens opacity.^2^

Given that protective equipment plays a vital role in safeguarding medical staff, it is important that their functionality be ensured, which includes confirming the specified lead equivalence,^5^ as this is an established standard of protective capability.^6^ This is particularly important for garments that consist of materials other than lead (lead‐free garments), as the protection offered can differ notably from that of lead, depending on the x‐ray beam quality.^7^, ^8^ Verification usually involves using an x‐ray tube to measure the dose reduction achieved with the protective equipment, followed by a comparison against that achieved with reference lead sheets of various thicknesses.^9^, ^10^, ^11^, ^12^, ^13^, ^14^, ^15^ Depending on the precision sought, numerous measurements with different lead sheets may be required, and this can be challenging to implement in a large medical facility with diverse protective equipment. In order for the testing method to be viable in a busy clinical environment, it is important to balance accuracy with practicality and expediency.

An efficient alternative is to use a computational model that translates the measured dose reduction into the thickness of lead necessary to achieve the observed attenuation. In this scenario, a single pair of measurements—one taken with and one taken without the protective equipment in the x‐ray beam—would be sufficient to determine the lead equivalence for a given beam quality. The purpose of the current study is to introduce and substantiate this methodology, utilizing a previously developed analytical spectrum model to simulate the incident beam. This includes exploring appropriate x‐ray tube beams for testing protective equipment by revisiting the previously introduced concept of scatter‐mimicking primary x‐ray beams, considering an extended range of scatter directions and highly filtered x‐ray tube beams typical in interventional radiology.

## 2 METHODS

### 2.1 Computational approach

Evaluating the lead equivalence of protective equipment begins with assessing how well it reduces incident radiation, which can be quantified by either transmission,

(1) $$T\left(Q\right)=\frac{K_{\mathrm{air},1}}{K_{\mathrm{air},0}},$$

or attenuation,

(2) A(Q)=1−T(Q),

where $K_{\mathrm{air},1}$ and $K_{\mathrm{air},0}$ represent the air kerma free‐in‐air measured with and without the protective equipment placed in an x‐ray beam of quality Q, respectively. These measures can be obtained using a standardized experimental setup, such as that outlined in the International Electrotechnical Commission (IEC) standard 61331‐1^6^ for assessing the attenuation properties of materials:

a. Narrow‐beam geometry is designed to measure the dose reduction of the incident x‐ray beam while considering only the primary incident x rays.
b. Broad‐beam geometry is designed to measure the dose reduction of the incident x‐ray beam, taking into account the contribution of secondary photons (fluorescence and Compton scattering) emitted from the material.

These geometries address different objectives. A narrow beam is suitable for verifying the inherent absorption of a material, while a broad beam is useful for determining the effective protection in typical clinical exposure scenarios. For instance, the importance of broad‐beam geometry has been recognized when evaluating lead‐composite or lead‐free garments, as they may emit a substantial amount of secondary photons in the form of K‐shell fluorescence, which reduces the protection.^7^, ^16^

It is important to distinguish between the dose reduction measured using a narrow or broad beam, as they differ in both concept and numerical value. To clarify this distinction, the transmission can be expressed in an expanded form, considering the primary (p) and secondary (s) components of the radiation. The narrow‐beam transmission for a material with a thickness of t can be formulated as follows:

(3) $$T_{N}\left(Q,t\right)=\frac{{\left(K_{\mathrm{air},1}\right)}^{p}}{{\left(K_{\mathrm{air},0}\right)}^{p}}=\frac{\int dk\;k{\left[\Phi_{k}\right]}_{\mathrm{air}}^{p}\;{\left[\mu_{\mathrm{en}}\left(k\right)/\rho \right]}_{\mathrm{air}}f\left(k,t\right)}{\int dk\;k{\left[\Phi_{k}\right]}_{\mathrm{air}}^{p}\;{\left[\mu_{\mathrm{en}}\left(k\right)/\rho \right]}_{\mathrm{air}}},$$

whereas the broad‐beam transmission can be represented in a factorized form,

(4) $$\begin{array}{cc} T_{B}\left(Q,t\right) & =\frac{{\left(K_{\mathrm{air},1}\right)}^{p+s}}{{\left(K_{\mathrm{air},0}\right)}^{p}}=\frac{{\left(K_{\mathrm{air},1}\right)}^{p}}{{\left(K_{\mathrm{air},0}\right)}^{p}}\left[\frac{{\left(K_{\mathrm{air},1}\right)}^{p+s}}{{\left(K_{\mathrm{air},1}\right)}^{p}}\right]\\ & =T_{N}\left(Q,t\right)\;B\left(Q,t\right). \end{array}$$

In these expressions, ${\left[\mu_{\mathrm{en}}\left(k\right)/\rho \right]}_{\mathrm{air}}$ is the mass energy‐absorption coefficient in air for photons of energy k, and ${\left[\Phi_{k}\right]}_{\mathrm{air}}^{p}$ is the primary photon spectrum (fluence differential in energy) evaluated free‐in‐air. The transmission of primary photons is accounted for by the factor f(k,t)=exp(−μ(k)t), which is based on the linear attenuation coefficient μ, depending on the photon energy and the material composition. In the broad‐beam case, factorization introduces the buildup factor, B(Q,t), which accounts for the addition of secondary photons emitted from the irradiated material.

The analytical expressions provided above indicate that the transmission (and, analogously, the attenuation) is not only measurable but also calculable. This establishes a framework for determining lead equivalence by comparing a measured transmission value with the transmission computed for a certain lead thickness. The computational approach, which involves a model for the incident photon spectrum, is detailed in the following section.

#### 2.1.1 Determining the lead equivalence

Having measured the dose reduction achieved with the protective equipment, its lead equivalence can be determined by substituting the protective equipment with lead sheets of varying thicknesses until an identical dose reduction is observed. Although this approach is rather straightforward, it entails modifications to the measurement setup to accommodate the lead sheets and requires multiple additional measurements for each beam quality considered to cover a range of lead thicknesses.

An efficient alternative that eliminates the need for handling lead sheets and minimizes the number of measurements required involves using a computational model that translates the dose reduction measured for a protective equipment into a corresponding lead equivalence. This approach can be developed based on Equations (3 and (4), using a spectrum model that accurately describes the incident (primary) x‐ray beam. By matching the transmission for lead computed with these equations to the transmission measured for a specific protective equipment, the lead equivalence can be determined. In practice, this can be accomplished through an algorithm that iteratively adjusts the lead thickness until the computed and measured transmission match. This is analogous to the common method of computing the aluminum half‐value layer (HVL) thickness by iteratively varying the thickness of aluminum until the transmission is halved.^17^, ^18^

The above‐described task of determining lead equivalence can be stated as finding the thickness t of lead that minimizes the difference between computed (“comp”) and measured (“meas”) transmission, respectively. In the case of a narrow beam, this can be formulated as follows:

(5) $$t_{N}=\underset{t}{\arg\min}\left|{\left(T_{N}^{\mathrm{pb}}\left(Q,t\right)\right)}_{\mathrm{comp}}-{\left(T_{N}^{x}\left(Q\right)\right)}_{\mathrm{meas}}\right|,$$

where the superscript “pb” indicates that the computed transmission is for lead, while “x” indicates that the measured transmission is for protective equipment of unknown material. In the case of a broad beam, the task can be expressed as follows:

(6) $$\begin{array}{cc} t_{B} & =\underset{t}{\arg\min}\left|{\left(T_{B}^{\mathrm{pb}}\left(Q,t\right)\right)}_{\mathrm{comp}}-{\left(T_{B}^{x}\left(Q\right)\right)}_{\mathrm{meas}}\right| \end{array}$$

(7) $$\begin{array}{cc} & =\underset{t}{\arg\min}\left|{\left(T_{N}^{\mathrm{pb}}\left(Q,t\right)\right)}_{\mathrm{comp}}-\frac{{\left(T_{B}^{x}\left(Q\right)\right)}_{\mathrm{meas}}}{B^{\mathrm{pb}}\left(Q,t\right)}\right|. \end{array}$$

These equations represent two interchangeable approaches to calculating the lead equivalence for a broad beam:

i. The approach described in Equation (6) involves the use of a computational model for broad‐beam transmission that explicitly accounts for the contribution of secondary photons emitted from a pure lead sheet. This can be realized as follows:
(8) $$T_{B}^{\mathrm{pb}}\left(Q,t\right)=T_{N}^{\mathrm{pb}}\left(Q,t\right)\;B^{\mathrm{pb}}\left(Q,t\right).$$
ii. The approach described in Equation (7) involves the use of a computational model for narrow‐beam transmission, and scaling the broad‐beam transmission measured for a protective equipment by the factor $B^{\mathrm{pb}}\left(Q,t\right)$, which is the ratio of broad‐ to narrow‐beam air kerma behind a pure lead sheet. This can be realized as follows:
(9) $$T_{N}^{\mathrm{pb}}\left(Q,t\right)=T_{B}^{\mathrm{pb}}\left(Q,t\right)\;/\;B^{\mathrm{pb}}\left(Q,t\right).$$

It is evident that, regardless of whether the lead equivalence is determined using a narrow or broad beam, a computational model that predicts narrow‐beam transmission can be utilized. Recall from Equation (3) that the core component of such an approach is the incident x‐ray spectrum, which can be effectively represented by an analytical spectrum model. In this work, the KQP model,^19^ integrated into the SpekPy toolkit,^20^ was chosen because of its close correspondence with detailed Monte Carlo (MC) simulations and experimentally measured x‐ray spectra.^21^

Besides modeling the incident spectrum, the SpekPy toolkit offers a function to determine the thickness of a material (such as lead) required to achieve a specific transmission. This function can be used to compute the lead equivalence by setting the transmission input to the value measured for the protective equipment being investigated. It is applicable for narrow beams (according to Equation 5), and it can be used for broad beams by scaling the measured transmission by the buildup factor for lead (according to Equation 7). In this work, recommended numerical values for this factor are provided based on the measurements described in Section 2.2.

The approach proposed in this work for estimating the lead equivalence can thus be reduced to performing a pair of air‐kerma measurements (with and without the protective equipment) combined with a few lines of Python code. The following code exemplifies how the lead equivalence can be determined, assuming a transmission of 10% for a tube voltage of 90 kV and an additional filter of 0.1‐mm Cu, measured using a narrow beam produced by an x‐ray tube with a 12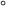 anode angle and inherent filtration of 3‐mm Al.




**import** spekpy as sp
*# the incident spectrum (90 kV, 12 deg anode angle)*:
s = sp.**Spek**(kvp=90,th=12,physics='kqp')

*# filter by 3.0 mm of Al and 0.1 mm Cu*:
s.**filter**('Al',3.0); s.**filter**('Cu',0.1)

*# compute the thickness of lead required for 10% transmission*:
t = s.**get_matl**(matl='Pb',frac=0.1)




### 2.2 Experimental verification

**FIGURE 1:**
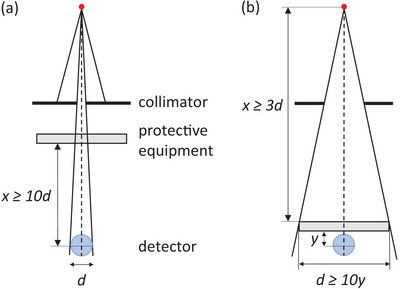
Geometries suitable for measuring the dose reduction of radiation protective equipment following IEC 61331‐1.^6^ Panel (a) illustrates narrow‐beam conditions and panel (b) illustrates broad‐beam conditions. In this study, the narrow‐beam geometry used x=150 cm and d=5 cm. For broad‐beam exposures, either (x=130, y=3, d=30) cm or (x=130, y=1, d=10) cm was used, depending on the specific measurement.

This section outlines the measurements performed to validate the computational model and to demonstrate its use in evaluating the lead equivalence accuracy of various radiation protective equipment. A Multitom Rax x‐ray system (Siemens Healthineers, Forchheim, Germany), equipped with a 12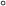 inclined tungsten anode tube, was employed for this task. The system incorporates lead collimators that were used to configure the narrow and broad beams illustrated in Figure 1. The beam qualities considered are specified in Table 1, encompassing the typical range of tube voltages and filters employed in interventional radiology.^22^ This is a more extensive range of qualities (in terms of HVL) than typically required to investigate protective equipment, as they represent primary beams rather than scatter beams relevant for evaluating radiation protection.^15^ Note, however, that scatter beams are also considered in this study, as covered in Section 2.3.

**TABLE 1:** x‐ray beams used to measure the dose reduction (transmission) of radiation protective equipment. For equipment with a specified lead equivalence exceeding 0.5‐mm lead, higher‐energy beams were used for reliable dose measurement behind the shielding material. The beams are characterized in terms of the tube voltage (kV), copper (Cu) filter thickness in millimeter (in addition to the 3 mm inherent Al filtration), the first half‐value layer thickness in millimeter of Al (HVL) and the ratio of first HVL to second HVL (h).

≤0.5 mm Pb				>0.5 mm Pb			
kV	Cu	HVL	h	kV	Cu	HVL	h
60	0.0	2.3	0.74	81	0.0	3.1	0.69
60	0.2	4.2	0.87	81	0.2	5.7	0.84
60	0.5	5.6	0.93	81	0.5	7.5	0.92
60	1.0	6.6	0.97	81	1.0	8.9	0.96
90	0.0	3.4	0.67	100	0.0	3.8	0.66
90	0.2	6.3	0.83	100	0.2	6.9	0.83
90	0.5	8.2	0.91	100	0.5	8.8	0.91
90	1.0	9.6	0.96	100	1.0	10	0.96
121	0.0	4.7	0.66	121	0.0	4.7	0.66
121	0.2	7.9	0.84	121	0.2	7.9	0.84
121	0.5	9.9	0.91	121	0.5	9.9	0.91
121	1.0	11	0.95	121	1.0	11	0.95

**FIGURE 2:**
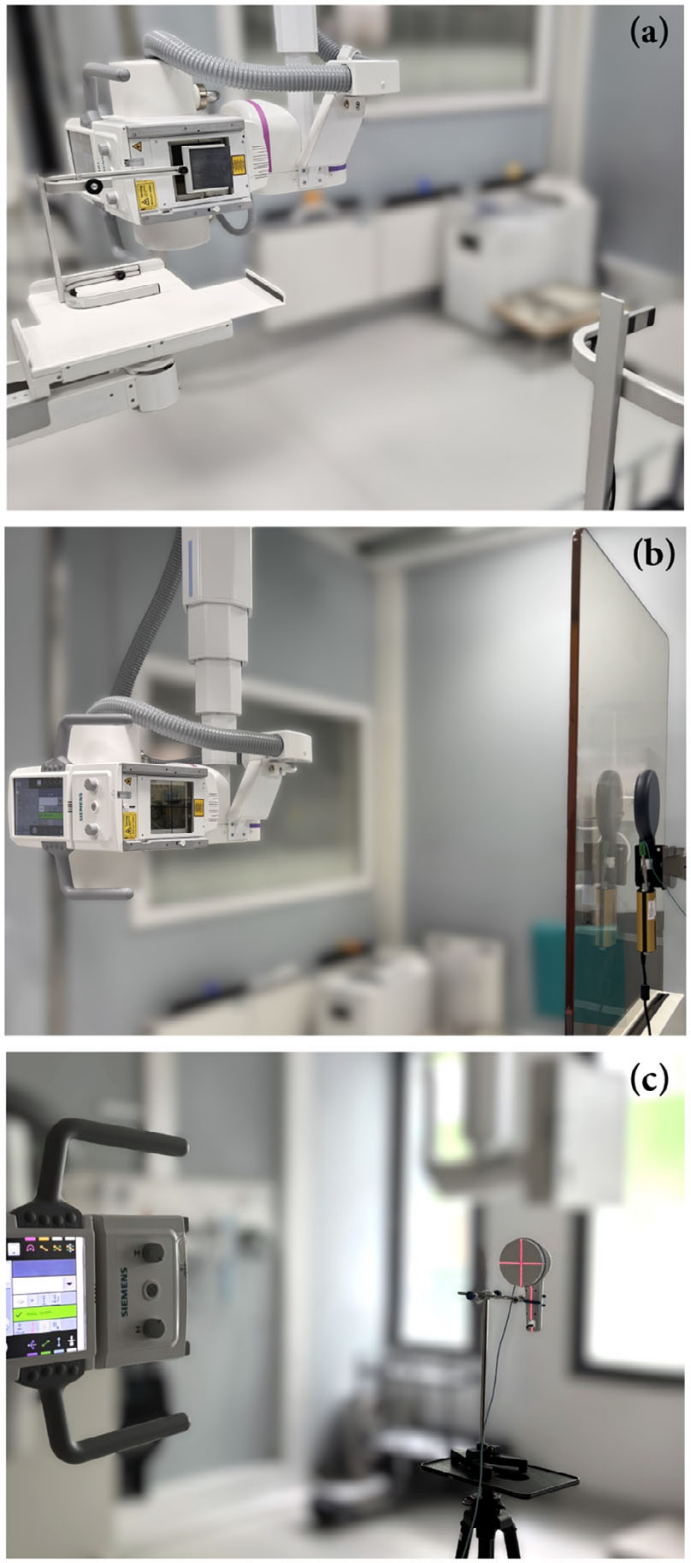
Images of the different measurement setups used: (a) narrow‐beam transmission for a lead sheet measured with the RTI Dose Probe, (b) broad‐beam transmission for an acrylic screen measured with the Radcal 10X6 180 ionization chamber, and (c) air kerma free‐in‐air assessed with the RaySafe 452 survey meter. In each image, the x‐ray tube of the Siemens Multitom Rax system is located on the left side, while the detector is situated on the opposite side.

The air kerma was measured with and without the protective equipment placed in the x‐ray beam using a solid‐state detector suitable for low‐dose‐rate measurements. The RTI Dose Probe (RTI Group, Mölndal, Sweden) consists of a silicon PIN photodiode coupled with a filter that reduces the inherent energy dependence. Despite this, a small energy correction (less than 4%) was applied using manufacturer‐supplied correction factors to account for differences in the spectra with and without the protective equipment. After applying this correction, the expanded uncertainty (k=2, corresponding to approximately a 95% confidence level) was below 3%. In order to investigate how the results may differ depending on the detector type used, additional measurements were performed with the Radcal 10X6 180 ionization chamber (Radcal, Monrovia, USA) and the RaySafe 452 radiation survey meter (Unfors RaySafe AB, Hovås, Sweden), which consists of a silicon sensor cluster combined with a Geiger–Müller pancake detector. These detectors were selected for their high sensitivity, linear response over a wide range of dose rates, and consistent energy response; as detailed in Table 2. Such attributes are necessary for accurate transmission measurements, as the dose is measured free‐in‐air as well as behind protective equipment (see Figure 2). All three detectors considered have calibration traceability to the Physikalisch‐Technische Bundesanstalt (PTB).

**TABLE 2:** Technical specifications for the different detectors used to evaluate the dose reduction (transmission) of radiation protective equipment.

Detector	Type	Sensitive area	Dose–rate range	Energy range
RTI Dose Probe	Diode	1 ${\mathrm{cm}}^{2}$	4 nGy/s–150 mGy/s	40–160 keV
Radcal 10X6 180	Ion chamber	100 ${\mathrm{cm}}^{2}$	50 nGy/s–4.9 mGy/s	30–1330 keV
RaySafe 452	Diode and GM	125 ${\mathrm{cm}}^{2}$	6 nGy/s–0.27 mGy/s	30–5000 keV

Table 3 outlines the various protective equipment investigated, including lead‐free aprons made of antimony (Sb) and bismuth (Bi).^23^ Furthermore, to substantiate the computational approach introduced, lead sheets from Goodfellow Cambridge Ltd. (UK) were employed. These sheets, measuring 10 by 10 ${\mathrm{cm}}^{2}$ and ranging in thickness from 0.1 to 1 mm, were supplied *as rolled* with a tolerance of ±10%; their thickness was verified using a micrometer screw gauge. The transmission values measured with these sheets were further used to derive $B^{\mathrm{pb}}\left(Q,t\right)$ entering Equation (7).

**TABLE 3:** Specified lead equivalence (${\mathrm{Pb}}_{\mathrm{eq}}$) for the different radiation protective equipment investigated, comprising personal protective aprons from Scanflex Medical AB (Täby, Sweden), an acrylic screen by MAVIG GmbH (Munich, Germany), and a custom‐made opaque screen from Skärmteknik (Norrköping, Sweden).

Type	Material	${\mathrm{Pb}}_{\mathrm{eq}}$
Personal apron	Lead free	0.25 mm
Personal apron	Lead free	0.35 mm
Personal apron	Lead vinyl	0.50 mm
Mobile screen	Lead acrylic	0.50 mm
Mobile screen	Lead plywood	1.00 mm

### 2.3 Monte Carlo simulation of clinical scatter beams

The suggested computational approach involves assessing the transmission of x rays through protective equipment by exposing it to radiation emitted directly from an x‐ray tube, as opposed to radiation scattered from a phantom. The scatter beam is expected to differ from the primary incident beam as a result of the Compton shift in energy and the attenuation of the scattered x‐rays. In order to better understand this effect, MC simulations of scatter photon fluence spectra have been performed using the PENELOPE MC system^24^ with the *penEasy* user code.^25^ The results of these simulations are intended to inform the selection of x‐ray tube parameters (tube voltage and filtration) to assess the lead equivalence of radiation protective equipment.

**FIGURE 3:**
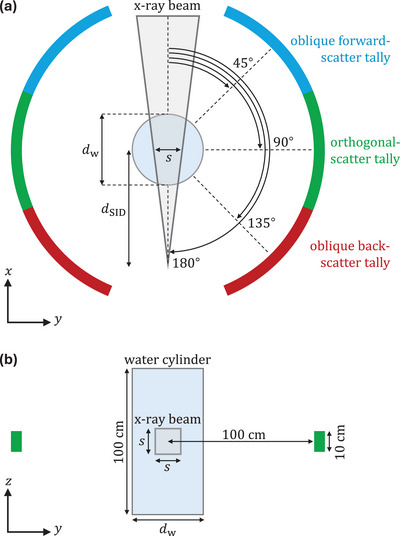
Geometry used for Monte Carlo simulation of scatter x‐ray spectra: (a) illustrates the xy‐plane, whereas (b) illustrates the yz‐plane. The configuration consists of a water cylinder with a diameter of $d_{w}=30$ cm exposed by a divergent x‐ray beam with a field size of s×s=10×10 ${\mathrm{cm}}^{2}$ at a source‐to‐isocenter distance of $d_{\mathrm{SID}}=80$ cm. Scattered x‐rays were tallied in the specified regions in air.

The simulation geometry consists of a 30‐cm water cylinder exposed to a 10 by 10 ${\mathrm{cm}}^{2}$ x‐ray beam, as illustrated in Figure 3. These parameters correspond to typical exposure settings (including the water‐equivalent thickness) encountered in interventional radiology.^22^ The x‐ray beam incident upon the water phantom was simulated using different spectra generated by SpekPy.^18^ These spectra served as source inputs for the user code, encompassing tube voltages of 60, 90, and 120 kV. The inherent filtration was assumed to be 3‐mm aluminum, to which 0.2, 0.5, 1.0 mm or no additional copper filtration was added. The particle transport in water was simulated with the electron transport kinetic energy cutoff set higher than the tube voltage (as radiative energy losses are negligible), while the photon energy cutoff was set to 10 keV.

The track‐length estimator was used to score the fluence differential in photon energy, considering different directions of scatter relative to the incident beam: oblique forward scatter at an angle of 45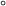, orthogonal scatter at 90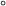, and oblique back scatter at 135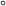. The fluence was tallied in 1‐keV energy bins with a statistical uncertainty of less than 0.5%.

#### 2.3.1 Determining x‐ray tube parameters to replicate scatter beams

The MC simulated spectra were used to identify x‐ray tube parameters that are suitable for replicating clinically relevant scatter beams. This was achieved through numerical optimization, which aimed to match the scatter spectra with the primary x‐ray tube spectra calculated using SpekPy.^18^ The optimization algorithm involved adjusting the tube voltage (ranging from 40 to 120 kV in 1‐kV increments) and copper filtration (ranging from 0 to 1 mm in 0.1‐mm increments) until agreement was achieved. The target criterion for acceptable agreement was that the first, second, and the combined first and second tenth‐value layer (TVL) thicknesses in lead had to match within a 5% margin. Recall that the first TVL thickness (${\mathrm{TVL}}_{1}$) corresponds to 10% transmission, whereas the combined first and second TVL thickness (${\mathrm{TVL}}_{1+2}$) corresponds to 1% transmission. When multiple combinations of tube voltage and filtration satisfied this criterion, the configuration with the least amount of copper filtration and the lowest tube voltage was chosen to minimize the resulting tube load.

The concept of scatter‐mimicking primary x‐ray beams to evaluate protective equipment was pioneered in previous research,^26^, ^27^, ^28^ which influenced ASTM International (formerly known as the American Society for Testing and Materials) to adopt it into the ASTM F3094‐14 standard for testing radiation protective equipment.^15^ In these previous studies, spectra were matched according to the first or second HVL in aluminum or copper. However, in this work, the surrogate spectra are formulated on the basis of the TVL thickness in lead. Since ${\mathrm{TVL}}_{1}$ and ${\mathrm{TVL}}_{1+2}$ correspond to 10 and 1% transmission, respectively, they offer a realistic representation of the dose reduction provided by radiation protective equipment. Furthermore, by employing lead instead of aluminum or copper, the specific attenuation characteristics of lead, particularly its K edge, are explicitly accounted for. It should also be noted that in the present work, the scatter spectrum is analyzed not only at 90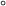, but also at larger (135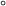) and smaller (45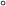) scatter angles. The spectrum at a larger angle could be relevant when assessing the effectiveness of table‐mounted shields or personal aprons in the case of lateral projection image acquisition. The spectrum at a smaller angle could be relevant when assessing the degree to which lead–acrylic screens or leaded glasses protect the eyes.

## 3 RESULTS

### 3.1 Experimental verification

#### 3.1.1 Lead sheets

**FIGURE 4:**
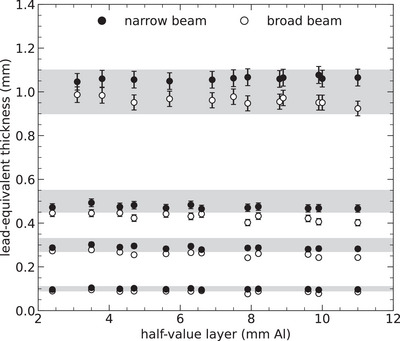
Lead equivalence determined for high‐purity lead sheets with specified thicknesses of 0.1, 0.3, 0.5, and 1 mm. These results were obtained through transmission measurements using narrow‐ and broad‐beam geometries. The x‐ray beam qualities used are detailed in Table 1. The uncertainty bars represent two standard deviations (coverage factor of k=2), and the gray bands signify the manufacturer's specified thickness tolerance.

In order to assess the accuracy of the proposed computational approach, it was used to determine the thickness of high‐purity lead sheets of known sizes. The results are presented in Figure 4, including the uncertainty of the measurements expressed as the combined relative standard uncertainty of ±4% (coverage factor of k=2). This includes the uncertainty related to the repeatability and reproducibility of the measurements, along with the uncertainty associated with the energy dependence of the detector. Note that this relative uncertainty extends to all subsequent results.

The figure presents two sets of results, one based on narrow‐beam transmission measurements and the other based on broad‐beam transmission. The narrow‐beam results demonstrate that the computational approach can predict the thickness of the lead sheets with an accuracy of 6%, which is within the manufacturer's thickness tolerance (as indicated by the gray band in the figure). However, it must be emphasized that the accuracy improves to approximately 3% (which is within the measurement uncertainty) if the computed thickness is compared to the average thickness assessed with a micrometer screw gauge. For example, the 1.0‐mm lead sheet was found to have an average thickness of 1.05 (±0.04) when measured with the micrometer screw gauge, confirming the slight overestimation in the computed thickness.

**FIGURE 5:**
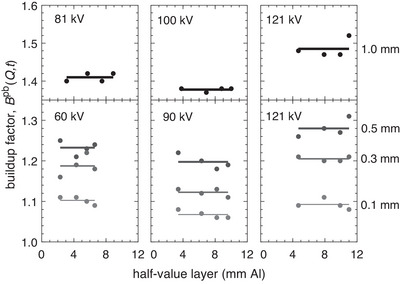
Buildup factor measured for different thicknesses of lead (0.1–1.0 mm, as indicated), which is used in Equation (7) for determining the lead equivalence based on broad‐beam transmission measurements. Note that the lines depict the average value for various combinations of lead thickness (t) and tube voltage (kV). The different x‐ray beam qualities (Q) considered are detailed in Table 1.

Notice that the lead thickness computed based on broad‐beam transmission measurements is lower than the corresponding result for a narrow beam, with a difference of 5%–28% depending on the beam quality. This discrepancy arises because the employed spectrum model does not consider secondary photon emission from the lead sheet, resulting in an underestimation of transmission for broad beams. However, as outlined in Equation (7), this limitation can be precisely accounted for by scaling the transmission by the buildup factor for lead, which corresponds to the ratio between the broad‐ and narrow‐beam air kerma evaluated behind lead. According to this definition, this factor has been empirically determined based on the performed transmission measurements and is presented in Figure 5.

The figure shows that the buildup factor varies from about 1.1 for a lead sheet that is 0.1‐mm thick to 1.5 for one that is 1.0‐mm thick. Additionally, note that the ratio is relatively consistent for specific combinations of lead thickness and tube voltage, exhibiting only a slight dependency on the additional beam filtration applied, as shown by the lines representing the average values. These results for the buildup factor are incorporated into subsequent computations of the lead equivalence based on broad‐beam transmission, as per Equation (7).

#### 3.1.2 Protective equipment

**FIGURE 6:**
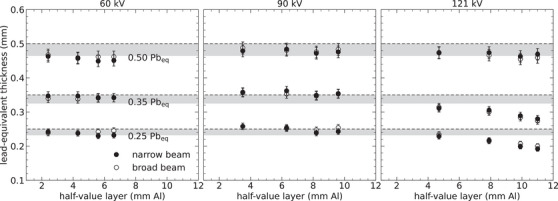
Lead equivalence accuracy determined for three personal aprons: two lead‐free aprons (0.25 and 0.35‐mm ${\mathrm{Pb}}_{\mathrm{eq}}$) and one lead–vinyl apron (0.5‐mm ${\mathrm{Pb}}_{\mathrm{eq}}$). These results were obtained based on measured narrow‐ and broad‐beam transmission, with the latter scaled by the buildup factor according to Equation (7). The x‐ray beam qualities applied are detailed in Table 1, with each panel containing measurements at the specified tube voltage (kV). The uncertainty bars represent two standard deviations (coverage factor of k=2), and the gray bands signify the minimum acceptable compliance range according to IEC.^6^

**FIGURE 7:**
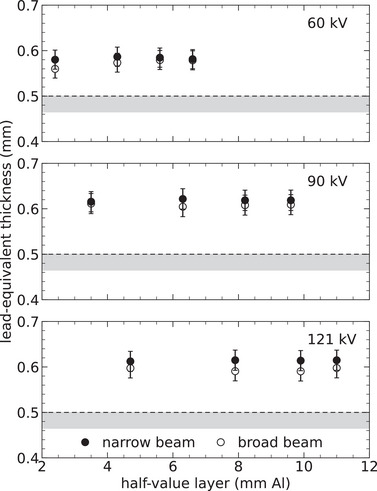
Lead equivalence accuracy determined for a lead‐acrylic mobile screen with specified thickness of 0.5 mm. These results were obtained based on measured narrow‐ and broad‐beam transmission, with the latter scaled by the buildup factor according to Equation (7). The x‐ray beam qualities applied are detailed in Table 1, with each panel containing measurements at the specified tube voltage (kV). The uncertainty bars represent two standard deviations (coverage factor of k=2), and the gray bands signify the minimum acceptable compliance range according to IEC.^6^

**FIGURE 8:**
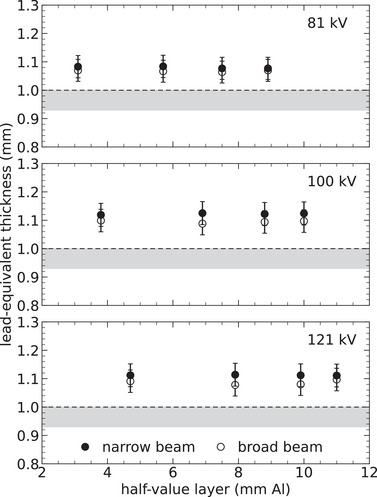
Lead equivalence accuracy determined for a lead‐plywood mobile screen with specified thickness of 1.0 mm. These results were obtained based on measured narrow‐ and broad‐beam transmission, with the latter scaled by the buildup factor according to Equation (7). The x‐ray beam qualities applied are detailed in Table 1, with each panel containing measurements at the specified tube voltage (kV). The uncertainty bars represent two standard deviations (coverage factor of k=2), and the gray bands signify the minimum acceptable compliance range according to IEC.^6^

Figures 6, 7, 8 present the lead equivalence accuracy for different types of protective equipment (specified in Table 3), evaluated based on transmission measurements for a wide range of beam qualities. The lead equivalence values determined are contrasted with the specified lead equivalence of each protective equipment, considering the minimum acceptable compliance range according to IEC ($\geq 0.93\;{\mathrm{Pb}}_{\mathrm{eq}}$).^6^

Figure 6 shows that the aprons meet the compliance criteria if the HVL for the incident beam is less than approximately 6 mm of Al. Notice, in particular, the reduced protection of the two lead‐free aprons (0.25‐ and 0.35‐mm ${\mathrm{Pb}}_{\mathrm{eq}}$) at high tube voltages, leading to a decrease in the lead‐equivalent thickness of up to 20%. The figure also demonstrates good agreement between the lead equivalence determined from both narrow‐ and broad‐beam transmission measurements, provided that the broad‐beam transmission has been scaled by the buildup factor $B^{\mathrm{pb}}\left(Q,t\right)$.

Figures 7 and 8 show that the lead‐equivalence of the two mobile screens remains consistent across various beam qualities. These screens not only meet but also markedly exceed the minimum compliance requirements for all beam qualities, whether using a narrow or broad beam. Nevertheless, their effectiveness in the latter case is reduced by up to 5%, suggesting that these screens produce somewhat more secondary photons compared to pure lead of equivalent thickness.

In order to investigate whether the choice of air‐kerma detector influences estimates of the lead‐equivalent thickness, the results for the lead–acrylic mobile screen presented in Figure 7 were re‐evaluated based on transmission measurements with two additional detectors. The small semiconductor photodiode (RTI Dose Probe) has thus been compared with a large‐volume ionization chamber (Radcal 10X6 180), and a survey meter incorporating a silicon sensor cluster combined with a Geiger–Müller pancake detector (RaySafe 452 Radiation Survey Meter). The findings, which are summarized in Table 4, demonstrate excellent agreement in lead equivalence, with a difference of less than 0.01 mm when using a narrow beam. A slightly higher discrepancy of 0.02 mm is observed when using a broad‐beam geometry. This can be explained by the larger sensitive areas of the Radcal and RaySafe detectors, which enable more secondary photons to be detected at steep incident angles, thereby increasing the measured transmission.

**TABLE 4:** Lead equivalence determined for a lead–acrylic mobile screen with specified thickness of 0.5 mm. These results were obtained through transmission measurements using different detectors (specified in Table 2), considering both narrow‐ and broad‐beam geometries. The results correspond to the average (min–max) for the x‐ray beam qualities detailed in Table 2.

	Lead equivalence (mm)	
Detector	Narrow beam	Broad beam
RTI Dose Probe	0.60 (0.58–0.62)	0.59 (0.56–0.61)
Radcal 10X6 180	0.60 (0.57–0.62)	0.57 (0.55–0.58)
RaySafe 452	0.59 (0.57–0.61)	0.57 (0.56–0.60)

### 3.2 Monte Carlo simulation of clinical scatter beams

**FIGURE 9:**
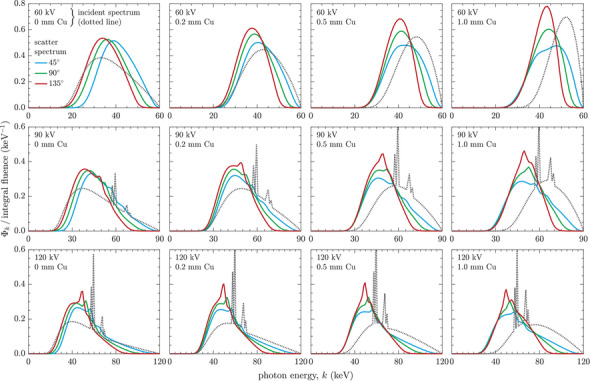
Scatter photon fluence spectra (fluence differential in energy normalized to the integral fluence) produced by various incident x‐ray beams specified by tube voltage (kV) and additional copper filter thickness (mm Cu). The incident spectrum was modeled using SpekPy,^20^ whereas the resulting scatter spectra are from Monte Carlo simulations implementing the geometry illustrated in Figure 3. Consideration is given to different scatter directions relative to the incident beam: oblique forward scatter (45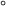), orthogonal scatter (90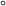), and oblique back scatter (135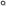).

Figure 9 illustrates the scatter spectra produced by MC simulations for various clinically relevant incident x‐ray beams. The beam quality of these scatter spectra is presented in Table 5, using the metrics of ${\mathrm{TVL}}_{1}$ and ${\mathrm{TVL}}_{1+2}$ in lead, representing the thickness of lead required for 10 and 1% transmission, respectively. These results serve as the basis for developing scatter‐mimicking primary x‐ray beams that are suitable for assessing the effectiveness of protective equipment.

**TABLE 5:** First and the combined first and second tenth‐value layer thickness in Pb (${\mathrm{TVL}}_{1}$ and ${\mathrm{TVL}}_{1+2}$), which signifies the lead thickness required for 10 and 1% transmission, respectively, for the photon fluence spectra presented in Figure 9. The spectra considered are the incident beam (in.), characterized in terms of the tube voltage (kV) and additional filter thickness in mm of Cu, along with the resulting scatter in diverse directions, as illustrated in Figure 3: oblique forward scatter (45), perpendicular scatter (90), and oblique back scatter (135).

		${\mathrm{TVL}}_{1}$ (mm Pb)				${\mathrm{TVL}}_{1+2}$ (mm Pb)			
kV	Cu	in.	$45^{\circ }$	90 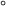	135 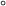	in.	45 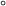	90 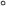	135 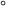
60	0.0	0.10	0.15	0.11	0.09	0.30	0.36	0.28	0.22
60	0.2	0.17	0.16	0.14	0.11	0.41	0.38	0.32	0.27
60	0.5	0.22	0.17	0.15	0.13	0.49	0.41	0.35	0.30
60	1.0	0.26	0.18	0.17	0.15	0.56	0.43	0.39	0.33
90	0.0	0.22	0.26	0.20	0.15	0.73	0.74	0.56	0.41
90	0.2	0.35	0.28	0.23	0.19	0.97	0.78	0.63	0.48
90	0.5	0.45	0.30	0.26	0.22	1.13	0.83	0.69	0.54
90	1.0	0.54	0.33	0.30	0.25	1.27	0.90	0.75	0.60
120	0.0	0.30	0.33	0.27	0.21	0.91	0.94	0.82	0.62
120	0.2	0.42	0.35	0.31	0.26	1.09	0.99	0.90	0.71
120	0.5	0.49	0.37	0.35	0.30	1.20	1.03	0.98	0.80
120	1.0	0.54	0.40	0.39	0.35	1.29	1.09	1.06	0.89

The figure shows that the scatter spectrum is generally softer (shifted to lower energies) compared to the incident spectrum, with ${\mathrm{TVL}}_{1}$ and ${\mathrm{TVL}}_{1+2}$ reduced by up to 54% (the average reduction being 25%). An exception to this pattern is observed for oblique forward scatter (45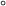) produced by incident x‐ray beams without additional copper filtration. In this case, the scatter spectrum is harder than the incident spectrum, resulting in an increase in ${\mathrm{TVL}}_{1}$ of up to 50% (average increase of 26%), while ${\mathrm{TVL}}_{1+2}$ is elevated by up to 20% (average increase of 8%). According to the results presented, the lead equivalence required to achieve 10% transmission may be overestimated by as much as 0.3‐mm Pb if clinically applied primary beams are considered instead of the resulting scatter beams. For 1% transmission, this overestimation can reach as much as 0.7 mm Pb.

Table 6 provides a list of x‐ray tube parameters suitable for replicating the quality of clinically relevant scatter beams, measured in terms of ${\mathrm{TVL}}_{1}$ and ${\mathrm{TVL}}_{1+2}$. These results indicate that the entire range of scatter‐mimicking primary beams can be achieved with tube voltages ranging from approximately 50 to 100 kV and a copper filter thickness of less than 0.3 mm. Specifically, the configurations (50 kV, 0.0‐mm Cu) and (100 kV, 0.2‐mm Cu) cover the range from the softest to the hardest scatter beam quality, with (75 kV, 0.1‐mm Cu) being an appropriate intermediary. Subsequently, narrow‐ and broad‐beam transmission measurements using these specific beam qualities were performed to reassess the lead equivalence accuracy of the personal aprons depicted in Figure 6. For these scatter beam qualities, the apron with a specified lead equivalence of 0.25 mm demonstrated an actual thickness ranging from 0.22 to 0.25 mm, while the apron with a specified thickness of 0.5 mm demonstrated an actual thickness ranging from 0.45 to 0.50 mm. The lowest thickness values were observed for the softest scatter beam quality considered (50 kV, 0.0‐mm Cu). For the other beam qualities, the measured thicknesses were in better agreement with the specified values: 0.24–0.25 mm compared to 0.25 mm, and 0.47–0.50 mm compared to 0.5 mm. These findings indicate that the aprons typically exhibit a lower lead equivalence than stated, in line with previous results obtained from comparisons with lead sheets,^10^ and from transmission measurements.^29^

**TABLE 6:** x‐ray tube parameters that can be used to replicate the quality of the scatter beams produced by different incident x‐ray beams, specified in terms of tube voltage (kV) and additional copper filter thickness (mm of Cu). Consideration is given to different scatter directions relative to the incident beam: oblique forward scatter (45), perpendicular scatter (90), and oblique back scatter (135). These results were produced by matching x‐ray tube spectra with Monte Carlo simulated scatter spectra (see Figure 9), on the basis of the first and the combined first and second tenth‐value layer thicknesses (TVL) in lead, as listed in Table 5.

Incident			$45^{\circ }$ scatter		$90^{\circ }$ scatter		$135^{\circ }$ scatter	
beam		↦	surrogate		surrogate		surrogate	
kV	Cu		kV	Cu	kV	Cu	kV	Cu
60	0.0		56	0.2	51	0.1	52	0.0
60	0.2		58	0.2	53	0.2	51	0.1
60	0.5		60	0.2	53	0.3	51	0.2
60	1.0		61	0.2	56	0.3	52	0.3
90	0.0		83	0.1	73	0.1	62	0.1
90	0.2		86	0.1	77	0.1	64	0.2
90	0.5		83	0.2	76	0.2	66	0.3
90	1.0		86	0.2	77	0.3	69	0.3
120	0.0		96	0.1	86	0.1	75	0.1
120	0.2		101	0.1	92	0.1	82	0.1
120	0.5		94	0.2	90	0.2	82	0.2
120	1.0		102	0.2	99	0.2	83	0.3

## 4 DISCUSSION

This work presents an efficient method for assessing the lead equivalence of x‐ray radiation protective equipment. The method requires a single pair of measurements to determine the transmission of the protective equipment, which is then converted to a lead‐equivalent thickness using a computational model. This approach removes the need for comparative measurements against reference lead sheets, reducing both measurement effort and improving reliability, since thin lead sheets are susceptible to deformation.

The accuracy of the method was demonstrated by comparing the predicted thickness to the actual thickness of high‐purity lead sheets (verified using a micrometer screw gauge). The accuracy was found to be approximately 3%, which is well within the manufacturer's tolerance of 10% (typical tolerance for thin lead sheets). The method can be applied to estimate the lead equivalence determined from both narrow‐ and broad‐beam transmission measurements. For broad beams, it is necessary to scale the measured transmission by the buildup factor for lead, if the analytical spectrum model used does not account for scatter (see Equation 7). This factor is applicable to protective equipment made of lead and nonlead materials (such as antimony‐based composites), because it represents buildup in the reference material (lead), not the protective material. The buildup of the protective material is inherently captured in the measured broad‐beam transmission. Appropriate numerical values for the buildup factor for lead have been determined through measurements and are reported in this work.

In the proposed procedure, transmission is measured by exposing the protective equipment to radiation emitted directly from an x‐ray tube, rather than to the radiation scattered from a phantom. This substantially simplifies the setup and reduces the required tube load, making the method suitable for both laboratory and clinical environments. Appropriate x‐ray tube parameters (voltage and filtration) must, however, be selected to emulate the quality of the scattered radiation relevant in the context of radiation protection. These so‐called scatter‐mimicking x‐ray tube beams have previously been investigated for 90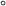 scatter produced by primary clinical beams with low filtration (up to 0.3 mm of additional copper).^26^, ^27^, ^28^ Here, the concept is extended to include both smaller and larger angles, relevant for evaluating protective equipment such as table‐mounted shields and ceiling‐suspended lead‐acrylic screens. Additionally, high‐filtration primary clinical beams with up to 1 mm of added copper, commonly used by modern cardiovascular and interventional imaging systems,^30^ have been considered.

Taking into account the factors mentioned, appropriate x‐ray tube parameters for testing protective equipment have been determined based on the transmission characteristics in lead for MC‐simulated scatter spectra. While Table 6 provides a complete list of these findings, Table 7 conveniently summarizes them into scatter‐mimicking beams indicative of low, moderate, and high transmission scatter, and compares these with the three beams specified in the ASTM F3094‐14 standard.^15^ The table also lists buildup factors for common protective lead thicknesses, which can be used with Equation (7) to estimate lead equivalence based on broad‐beam transmission measurements.

**TABLE 7:** Scatter‐mimicking beams suitable for testing protective equipment, characterized in terms of the tube voltage (kV), aluminum (Al) and copper (Cu) filter thickness in mm, and the first half‐value layer thickness in mm of Al (HVL). Also included are buildup factors $B^{\mathrm{pb}}\left(Q,t\right)$ estimated in this work for standard protective lead thicknesses, which can be used according to Equation (7) for evaluating the lead equivalence based on broad‐beam transmission measurements.

					$B^{\mathrm{pb}}\left(Q,t\right)$			
	kV	Al	Cu	HVL	0.25	0.35	0.5	1.0
This work	50	3.0	0.0	1.9	1.01	1.01	1.00	1.00
	75	3.0	0.1	4.3	1.12	1.16	1.21	1.43
	100	3.0	0.2	6.9	1.12	1.15	1.21	1.37
ASTM^15^	70	4.7^a^	0.0	3.4	1.13	1.17	1.23	1.42
	85	4.5^a^	0.0	4.0	1.13	1.16	1.22	1.40
	105	4.8^a^	0.0	5.1	1.13	1.17	1.22	1.40

a Inferred from the kV and HVL using SpekPy.^20^

The scatter‐mimicking beams introduced here differ from those recommended by ASTM mainly in two aspects. First, the beam filtration consists of standard copper filters in conjunction with a fixed aluminum filter thickness, reflecting the typical inherent filtration of clinical x‐ray tubes, which generally ranges from 2.5 to 3.5 mm in aluminum equivalence. This approach leverages the fact that many clinical radiography and radioscopy x‐ray systems have built‐in copper filters that can be used to achieve the desired scatter‐mimicking beams. Second, the beams cover a broader range of scatter spectra to address the diverse radiation protection scenarios faced in modern diagnostic and interventional radiology. Applications include assessing acrylic screens for eye lens protection, determining the required thickness of protective aprons for staff using angiography systems with substantial copper filtration, and verifying mobile screens and table‐mounted shields in operating rooms equipped with radioscopy and cone‐beam computed tomography (CBCT).

The proposed computational approach for evaluating lead equivalence has been demonstrated in this work for various kinds of protective equipment (see Figures 6, 7, 8), with consistent results achieved using different radiation detectors (see Table 4). In interpreting these results, it should be noted that the same procedure (identical geometries and beam qualities) was used to measure transmission through high‐purity lead sheets to validate the computational method, as was employed for testing the protective equipment. Consequently, given the high accuracy demonstrated by this validation, the computational results for the protective equipment can be considered equivalent to those that would be obtained with the more laborious conventional method for determining lead equivalence, which relies on comparison against reference lead sheets of varying thicknesses.

It is important to highlight that the accuracy of the proposed method is strongly dependent on the accuracy of the spectrum model used, which in turn depends on how well the assumed x‐ray tube parameters reflect the actual operating conditions. If the tube parameters are incorrectly specified, the calculated lead equivalence is rendered unreliable. This can, however, be mitigated by confirming the x‐ray beam quality through established standard quality control testing of x‐ray tubes. This includes checking the accuracy of the tube voltage and the thicknesses of both the inherent and additional filtration, for example by determining the aluminum HVL.

Another limitation involves the buildup factor $B^{\mathrm{pb}}\left(Q,t\right)$ used for assessing lead equivalence based on broad‐beam transmission measurements. The values provided are derived from measurements that inherently involve uncertainties and are specific to the settings used. Consequently, the factors listed apply only to the beam qualities for which they were determined, and their associated uncertainty is linked to the overall measurement uncertainty, which encompasses elements such as variations in the thickness of the high‐purity lead sheets, the type of detector employed, and the measurement geometry. A more general strategy would be to incorporate the buildup factor directly into the computational model (according to Equation 6) by accounting for the contribution of secondary photons emitted from pure lead sheets using detailed MC simulations.^31^, ^32^ This will be explored in future work, where the particular measurement conditions relevant to testing radiation protective equipment should be considered.

Regarding the broader applicability of the computational framework introduced, it should be noted that although the focus of this work has been on evaluating lead equivalence based on transmission in terms of air kerma free‐in‐air, the methodology can also be applied to other quantities. For instance, it has been suggested that the transmission may be evaluated in terms of the personal dose equivalent at a depth of 10 mm, $H_{p}\left(10\right)$.^28^ This could be accommodated simply by evaluating the transmission using a detector that measures $H_{p}\left(10\right)$, and in the computations, replacing the air kerma evaluation in Equation (3) with that of $H_{p}\left(10\right)$ using published conversion coefficients.^33^ Furthermore, the computational framework is not limited to the specific examples in this study related to the lead equivalence of protective equipment. It is readily applicable to a wide range of testing scenarios, including measurements with primary or scatter‐mimicking x‐ray tube beams, a variety of anode materials and anode angles, as well as different filter materials and tube voltages. Moreover, it can be employed to assess equivalence in terms of other commonly used shielding materials, such as concrete, steel, and gypsum wallboard.

## 5 CONCLUSIONS

A straightforward technique for evaluating the lead equivalence of x‐ray radiation protective equipment has been proposed and validated. This method involves converting the measured dose reduction (transmission) of protective equipment into lead equivalence using a computational model, which effectively eliminates the need to handle reference lead sheets. The computational process has been outlined, addressing both narrow‐ and broad‐beam transmission measurements and clarifying the different factors involved.

As part of this work, a simple implementation of the method has been realized using the freely available SpekPy toolkit^20^ for analytical x‐ray spectrum modeling; the few lines of code needed are included in the text. Furthermore, the concept of scatter‐mimicking x‐ray tube beams suitable for evaluating the effectiveness of protective equipment has been revisited, extending the scope to include various scatter angles and high‐filtration primary clinical beams frequently used in modern cardiovascular and interventional imaging. These findings, together with the outlined computational approach, offer a comprehensive framework for the efficient evaluation of the lead equivalence of protective equipment.

## CONFLICT OF INTEREST STATEMENT

The authors declare no conflicts of interest.
